# Utility of CD34 in Assessing Microvessel Density and Its Correlation With Clinicopathological Parameters in Colorectal Carcinoma Patients

**DOI:** 10.7759/cureus.49186

**Published:** 2023-11-21

**Authors:** Shweta Pandey, Samarth Shukla, Sunita Vagha

**Affiliations:** 1 Pathology, Jawaharlal Nehru Medical College, Datta Meghe Institute of Higher Education and Research, Wardha, IND

**Keywords:** perineural invasion, lymphovascular invasion, immunohistochemistry, cd34, mean vessel density, colorectal carcinoma

## Abstract

Currently, the most commonly practiced method of reporting cases of colorectal carcinoma is done according to guidelines provided by the College of American Pathologists (8^th^ edition) and the Royal College of Pathologists (UK). These guidelines include various histopathological parameters like tumor site, extent, histologic type, grade, margins, tumor budding, lymphovascular invasion, and perineural invasion. However, in the present guidelines, the immunohistochemistry-based marker of mean vessel density (MVD) has not been addressed as an important parameter.

The present study gives an overview of the importance of MVD. MVD was statistically significant when correlated with tumor size, lymph node metastasis, grade, and vascular invasion. However, no statistical significance was observed when compared with age, perineural invasion, and stage of the tumor.

## Introduction

Colorectal carcinoma (CRC) is one of the most common malignancies worldwide. Many developing countries have shown a significant rise in the incidence of colorectal malignancies. This is mainly ascribed to certain changes in modifiable factors like dietary patterns of increased consumption of meat products and also due to various lifestyle misconducts like obesity and lack of exercise [1,2]. More than 1.9 million new cases of CRC were reported in 2020, and there have been nearly 5,76,858 deaths from colon carcinoma and 3,39,022 deaths from rectal carcinoma, which is almost 9.4% of all cancer-related deaths, third only to breast and lung cancer [1,2].

Sustained tumor angiogenesis is one of the fundamental steps in tumor growth, invasion, and metastasis. Just like any other normal cell in the body, tumor cells also require the formation of a new vascular supply for nutrition and oxygenation [3-5], and hence an increase in the number of blood vessels in a tumor is significant for tumor growth and its metastatic potential [4]. Mean vessel density (MVD) is a biological parameter that can be utilized to determine angiogenesis and understand tumor behavior and biology [6-10]. MVD can be implied to be associated with a higher grade of tumors, metastasis, and neural invasion. CD34 is a very commonly employed immunohistochemical marker for endothelial cells and hematopoietic progenitor cells. The endothelial cells lining the blood vessels immunolabeled with CD34 can be used to evaluate the MVD [11-17].

The present study would like to propose the significance of MVD as a potential parameter in the reporting guidelines as it can prove to be exceedingly useful in analyzing tumor behavior and morphology.

## Materials and methods

The present study is a retrospective analytical study conducted over a period of one year and performed in the Department of Pathology, Jawaharlal Nehru Medical College, Sawangi (Meghe), Wardha, India, in collaboration with the Department of Oncosurgery, Acharya Vinoba Bhave Rural Hospital, Sawangi (Meghe), Wardha, India. The Institutional Ethics Committee gave their approval for the study (DMIHER(DU)/IEC/2023/650). A total of 100 cases diagnosed with carcinoma of the colon or rectum and had subsequently undergone hemicolectomy, total colectomy, low anterior resection, or abdominoperineal resection were included in the study. The sample size was calculated using Cochran's formula with an incidence rate of CRC of 19.5% [2] and the desired error of margin was 8%. The sampling technique used was purposive sampling. Cases confirmed as colorectal adenocarcinoma on histopathology, all subtypes of adenocarcinoma, were included in the study. Patients of all age groups and both genders were included in the study. Patients with recurrent carcinoma, patients on chemotherapy or radiotherapy, and biopsies were excluded from the study. The effect modifiers like age were controlled through stratification.

**Figure 1:**
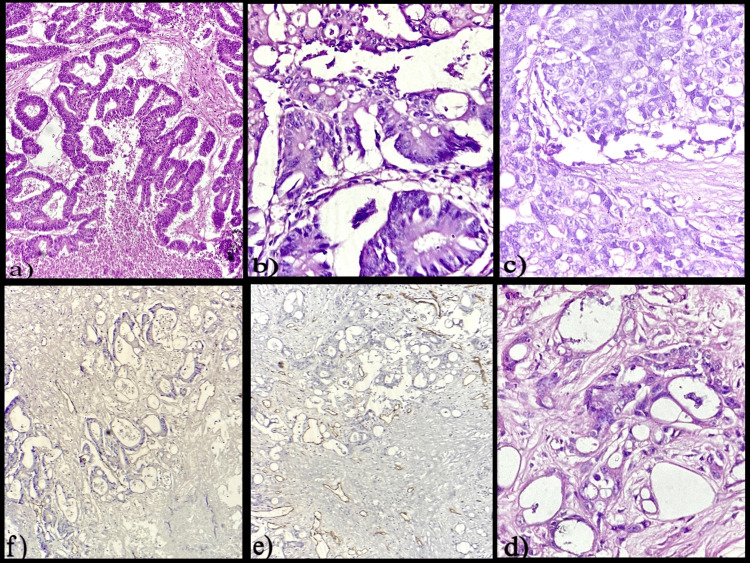
(a) Well-differentiated adenocarcinoma (H&E, low power view, 10x). (b) Moderately differentiated adenocarcinoma (H&E, 40x). (c) Poorly differentiated adenocarcinoma (H&E, 40x). (d) Section showing lymphovascular invasion in a moderately differentiated adenocarcinoma colon. (e & f) Section stained with IHC stain showing membranous positivity for CD34 in endothelial cells in well-differentiated adenocarcinoma colon (e) and moderately differentiated adenocarcinoma colon (f) (low power view, 100x) IHC: immunohistochemistry

Histopathological sections of tumor mass, margins, and lymph nodes from these resected specimens were studied, and tumor tissue was graded as well differentiated (Figure 1a), moderately differentiated (Figure 1b), or poorly differentiated (Figure 1c), and lymphovascular invasion (Figure 1d), perineural invasion, lymph nodes, margins status, and staging was done according to the pathological TNM staging provided by guidelines from the College of American Pathologists [4].

Calculation of MVD

Immunohistochemistry (IHC) staining was performed on these paraffin-embedded sections with vascular hot spots using CD34 (Figure 1e-1f). Sections with no or less necrosis, inflammation, or ulceration and the presence of angiogenesis were taken as vascular hot spots. CD34 expression was mainly detected on the cell membrane, in the cytoplasm, or in the tumor stroma. Immunolabeled vessels in at least three hot spots were counted under 400x magnification, and the average was considered the microvessel count/Hpf. Even in the absence of a vessel lumen, any staining in endothelial cells or cell clusters was considered one vessel.

Statistical tests

Statistical analysis was done by using descriptive and inferential statistics using Student’s unpaired t-test, one-way ANOVA, and Pearson correlation coefficient, and the software used in the analysis was SPSS Statistics version 27.0 (IBM Corp. Released 2020. IBM SPSS Statistics for Windows, Version 27.0. Armonk, NY: IBM Corp.), and p<0.05 is considered as the level of significance.

## Results

In the present study, the mean age was 54.35±12.98, and the maximum cases were seen in age groups above 50 years (62/100). The age of the patients when correlated with CD34 mean vessel count/Hpf was nonsignificant (0.341, NS) (Table 1). A slight male predominance of 53% was seen in cases of CRC, whereas 47% of affected patients were females. The maximum CD34-stained microvessel count observed was 42 and the minimum was 7 with the mean being 23.98 (Table 2). In the present study, the maximum cases were seen in the T3 category (38%), closely followed by 34% cases in the T2 category, and the least number of cases was observed in the T1 category with 8% cases. The maximum mean CD34 count was seen in T3 tumors (25.71). The study observed a significant correlation between the tumor extent (0.008, S), the grade of the tumor (0.0001, S), and the mean CD34 count.

**Table 1:** Correlation between CD34-stained microvessel count and parameters of tumor (Pearson correlation coefficient) NS: nonsignificant, S: significant

Variables	r-value	p-value
Age in years	-0.096	0.341, NS
Tumor extent	0.209	0.037, S
Lymph node involvement	0.633	0.0001, S
Vascular invasion	-0.658	0.0001, S
Perineural invasion	-0.451	0.25, NS
Grade of tumor	0.482	0.0001, S
Stage of tumor	0.419	0.057, NS

**Table 2:** Distribution of patients according to CD34-stained microvessel count

	N	Minimum	Maximum	Mean	Std. deviation
CD34 count	100	7.00	42.00	23.98	8.12

About 44% of cases showed no involvement of lymph nodes by the tumor cells and 66% of cases showed lymph nodes with positive tumor deposits on microscopy. The correlation between lymph node metastasis and CD34 count was significant (0.0001, S). In the present study, vascular invasion was identified in 39% of cases and vascular invasion was absent in 61% of cases. A significant correlation was observed between CD34 count and vascular invasion (0.0001, S). Tumor cells surrounding a nerve fiber are considered positive perineural invasions and are often associated with poor prognosis. In this study, perineural invasion was identified in 22/100 cases, and it was absent in 78/100 cases. The present study observed no significant correlation between perineural invasion and CD34 count (0.25, NS). The mean CD34 count in cases with perineural invasion was only slightly higher than in cases with absent perineural invasion. The correlation between the stage of tumor in the patients and their CD34 count was not significant (0.057, NS) (Table 1).

## Discussion

CRC ranks third with respect to incidence (29 per 100,000) and is second in terms of mortality in countries with higher human development indexes [2]. More than 9,35,000 deaths and more than 1.9 million new cases were reported in 2020 [2]. Like most other neoplasias, the diagnosis of CRC is also based on clinical, radiological, and pathological assessment. The risk of CRC is higher in first-degree relatives with CRC which increases with age. The most commonly inherited CRC syndrome is Lynch syndrome, also known as hereditary non-polyposis colorectal cancer and familial adenomatous polyposis. Mainly, the mutations observed in Lynch syndrome are in the DNA mismatch repair genes MLH1, MSH2, MSH6, and PMS2. Tumor angiogenesis is one of the hallmarks of cancer and a parameter that can be utilized in providing targeted therapy with respect to the proliferating vessels. Hence, the present study was carried out with the objective of assessing angiogenesis by calculating MVD (mean vessel count/Hpf) using the immunohistochemical marker CD34. The microvessel count was calculated by IHC using the CD34 marker. The CD34 values in the present study ranged from 7 to 42 microvessel/Hpf with the mean being 23.98/Hpf. The mean microvessel count of various studies is given in Table 3.

**Table 3:** Mean microvessel count in various studies

S. No.	Study and year	Mean microvessel count
01	Sharifi et al. [11] 2008	28.5/Hpf
02	Svagzdys et al. [8] 2009	193 + 11.2/mm2
03	Moreira et al. [16] 2011	35.75/Hpf
04	Deliu et al. [5] 2015	351.85/mm2
05	Toma et al. [14] 2018	304.6/mm2
06	Chabowski et al. [17] 2018	64.69 + 33.07/Hpf
07	Present study	23.98/Hpf

The present study observed no significant correlation between CD34 and the age of the patients (p-value=0.341). This is in agreement with the studies of Sheikh et al. [9] (0.291, NS), Deliu et al. [10] (0.704, NS), Sharifi et al. [11] (0.127, NS), Behebani et al. [12] (0.20, NS), and Qasim et al. [15] (NS).

The present study showed a significant correlation between CD34 expression and the extent of the tumor (0.008, S). This is in concordance with the study of Behebani et al. [12] (0.03, S) but contrary to the study of Sheikh et al. [9] (0.927, NS). The present study observed metastasis of CRC to lymph nodes in 54% of cases. The distribution of CD34 with lymph node metastasis in the present study showed a significant correlation (p-value=0.0001, S) which is in concordance with the study of Behebani et al. [12] (0.03, S) and discordant with the study of Sheikh et al. [9] (0.464, NS). Studies conducted by Sharifi et al. [11] (0.00, S) and Behebani et al. [12] (0.03, S) showed a significant correlation between CD34 expression and the grade of the tumor which is in agreement with the present study (0.0001, S). However, studies by Deliu et al. [10] (0.436, NS) and Sheikh et al. [9] (0.173, NS) are in disagreement with the present study. Lymphovascular invasion was identified in 39 cases of CRC. The correlation between lymphovascular invasion and CD34 expression was significant (0.00001, S) in the present study which is in concordance with the study of Behebani et al. [12] (0.01, S). Perineural invasion was identified in 22 cases of CRC. The present study observed no statistical significance with CD34 expression (0.25, NS) which is in disagreement with Behebani et al. [12] (0.03, S). In the present study, staging was done according to the American Joint Committee on Cancer guidelines (8^th^ edition). The majority of cases were seen in Stage III. The study observed no correlation between the stage of tumor of the CRC cases and CD34 expression (0.057, NS) which is similar to the study of Sharifi et al. [11] (0.544, NS), Qasim et al. [15] (NS), and Chabowski et al. [17] (0.4822, NS). However, it was contrary to the study of Deliu et al. [10] (p-value <0.01, S).

The limitations of the present study were technical as well as subjective. IHC is a technique that requires technical expertise and can be very expensive, and improper tissue sampling and tissue heterogenicity can lead to altered staining of CD34 immunohistochemical marker. Apart from IHC, one of the major restraints of this study is that the CD34 biomarker does not have a cut-off value and hence can affect the results. The duration of the study and the small sample size are also major limitations of the present study. Therefore, more follow-up studies are needed to document the correlation between MVD and clinicopathological parameters of CRC.

## Conclusions

The prognosis of colorectal cancer is associated with several clinical and pathological parameters. Age, sex, tumor size, tumor location, tumor multiplicity, tumor edge, tumor budding, margins, vascular invasion, perineural invasion, lymph node involvement, microscopic type, and tumor angiogenesis are a few of them. Microvessel density as a parameter suggests the blood vessel proliferation in a tumor. It can be useful for intratumoral quantification in CRC and also reflect on the tumor size, grade, lymph node involvement, and lymphovascular invasion.

The present study gave an overview of the importance of MVD. When correlated with clinicopathological parameters, MVD was statistically significant with tumor size, lymph node metastasis, grade, and vascular invasion. However, it showed no statistical significance when compared with age, perineural invasion, and stage of the tumor. The study suggested that when it comes to blood vessel proliferation and the metastatic tendency of a tumor, CD34 can prove to be a useful tool and provide better diagnosis and patient care in cases of CRC.
